# Fluorescent sensors for intracellular signaling in the brain: imaging neurons, glia, and vascular cells

**DOI:** 10.1117/1.NPh.12.S2.S22809

**Published:** 2025-11-11

**Authors:** Minkyung Kim, Manuel F. Navedo, Cam Ha T. Tran

**Affiliations:** a University of Nevada Reno, School of Medicine, Department of Physiology and Cell Biology, Reno, Nevada, United States; b University of California Davis School of Medicine, Department of Pharmacology, Davis, California, United States

**Keywords:** calcium, voltage, optical imaging, cAMP, brain imaging

## Abstract

Optical imaging has increasingly become the go-to technique for studying brain activity. The advancement of such approaches, which typically assess brain activity by monitoring the release or activity of second messengers, neurotransmitters, or electrical signals, has been entirely dependent on the development of sensors. Given advances in the field, a review of sensor development, including the latest sensors, is both timely and important for understanding their application in optical imaging. We seek to provide an overview of the sensors most commonly used by investigators to study brain function through optical imaging, including ${\mathrm{Ca}}^{2+}$, voltage, and cAMP sensors, highlighting their developmental trajectory, applications, and relative strengths and weaknesses. We systematically reviewed the most recent publications that describe either the development or use of optical sensors in the context of brain imaging. We evaluated technical specifications and performance in real-life applications of these biosensors. We identified and highlighted sensors that have been characterized and widely adopted in various applications. We discussed their utility, kinetics, and practical advantages and disadvantages. Because of their more advanced development, ${\mathrm{Ca}}^{2+}$ sensors receive more extensive consideration in our discussion. Overall, we reveal a plethora of available sensors that allow investigators to examine brain activity based on ${\mathrm{Ca}}^{2+}$ dynamics, cAMP activity, and electrical activity. Although further development is needed, the substantial progress in optical imaging, which is critically enabled by advances in sensor technology, is evident. These tools collectively provide researchers with powerful new capabilities to visualize and dissect the complex dynamics of brain function.

## 1 Introduction

The brain is a metabolically active organ that accounts for over 20% of the human body’s energy use, despite comprising only 2% of body mass. The brain not only consumes considerable energy, but also its metabolic demands vary from moment to moment and region to region. The 3-tier angioarchitecture of the brain vasculature, consisting of surface pial vessels, penetrating arterioles, and a vast capillary network, ensures that cerebral blood flow is regulated and distributed to every cell in the brain.^1^ Cerebral autoregulation ensures that the brain receives a constant blood supply sufficient to maintain basal activities despite changes in blood pressure.^2^^–^^6^ However, because the blood supply to the brain is limited, regionally varying needs cannot be satisfied by raising blood flow to every brain region simultaneously. Instead, there are mechanisms that ensure that the blood supply matches moment-to-moment changes in metabolic demands. Specifically, as neurons become more active, they require an increased delivery of oxygen and glucose to support their increased energy demands. This use-dependent increase in blood flow, known as functional hyperemia, is achieved through a coordinated process termed neurovascular coupling (NVC).^7^^–^^16^ Whereas cerebral autoregulation operates based on the intrinsic ability of vascular smooth muscle cells to respond to increased or decreased blood pressure by constricting or dilating, respectively,^2^^,^^4^^,^^17^^–^^20^ NVC operates by integrating components of the neurovascular unit, comprising neurons, astrocytes, and vascular cells.^8^^,^^9^^,^^11^^,^^12^^,^^15^^,^^21^^–^^31^ This interconnection of brain metabolism, neuroenergetics, and NVC ensures adequate blood flow to support neuronal activity and meet the brain’s changing energy needs. Neurovascular uncoupling has been implicated in a range of neurological disorders from ischemic stroke to Alzheimer’s disease,^30^^,^^32^ underscoring the pivotal importance of this regulatory mechanism. Researchers have made substantial progress in understanding NVC, and a significant contributor to these insights is optical imaging, which has increasingly become a key approach for studying the NVC process. Advancements in optical imaging modalities, which are closely tied to the development of sensors, provide unprecedented insights into intracellular signaling events within neurons, astrocytes, and vascular cells. The intrinsic symbiotic relationship between optical imaging modalities and sensors is the foundation for the advancement of our understanding of brain activity, including NVC. Although NVC inherently involves cell-to-cell communication, our emphasis here is on the intracellular signaling cascades within individual cell types that contribute to the NVC process.

Single-fluorophore and FRET-based sensors represent 2 fundamental designs used to monitor intracellular signals such as ${\mathrm{Ca}}^{2+}$, voltage, and cAMP. Single-fluorophore sensors typically rely on changes in fluorescence intensity of a single fluorescent protein or dye upon binding or detecting their target molecule or voltage change. By contrast, FRET-based sensors operate via Forster Resonance Energy Transfer between two fluorescent proteins or dyes, where binding of the target molecule or voltage change induces conformational shifts that alter FRET efficiency. This results in a ratiometric change in fluorescence emission from the donor and acceptor fluorophores. Both sensor types have been widely adapted and optimized for a range of intracellular signaling molecules, each offering unique benefits depending on the experimental requirements.

Here, we systematically review publications that discuss the development and/or use of fluorescent indicators, primarily ${\mathrm{Ca}}^{2+}$ indicators, and to a lesser extent, voltage and cAMP sensors, to study intracellular signaling events in the brain. Although these tools have been widely used to probe neuronal, glial, and vascular activity, we also highlight their current use and potential for investigating NVC.

## 2 Ca^2+^ Sensors

${\mathrm{Ca}}^{2+}$ is a vital, ubiquitous second messenger that is involved in almost every physiological process.^33^ In the brain, ${\mathrm{Ca}}^{2+}$ acts as a key signaling molecule, influencing neuronal activity, synaptic plasticity, and the transmission of information between cells, all of which are fundamental processes for cognitive functions and overall brain health.^34^ Astrocytes—specialized glial cells—tile the brain and are uniquely positioned to communicate with both neurons and the vasculature. Astrocytes respond to synaptic activity with a transient increase in intracellular ${\mathrm{Ca}}^{2+}$ that influences neurotransmitter uptake, ion balance, and gliotransmitter release and thereby impacts neuronal function.^35^ Astrocytes communication with both neurons and vascular cells can also take the form of the release of vasoactive agents that elicit vasodilation^36^^,^^37^ and, in certain cases, vasoconstriction.^38^^–^^40^ Changes in ${\mathrm{Ca}}^{2+}$ in vascular smooth muscle cells or endothelial cells play key roles in regulating vascular tone.^41^^,^^42^ Given the critical role of ${\mathrm{Ca}}^{2+}$ in diverse intracellular signaling pathways across all cell types in the brain, understanding ${\mathrm{Ca}}^{2+}$ signals, and their temporal and spatial characteristics, is essential to our understanding of brain activity, including the physiological regulation of NVC and the development of therapeutic approaches. The ideal ${\mathrm{Ca}}^{2+}$ signal-detection method should be specific, responding to ${\mathrm{Ca}}^{2+}$ signals and not others; sensitive, capable of detecting low concentrations of ${\mathrm{Ca}}^{2+}$; fast, with a temporal resolution sufficient to detect ${\mathrm{Ca}}^{2+}$ signals that occur prior to the physiological process under investigation; quantifiable; and minimally invasive. Although several methods for measuring ${\mathrm{Ca}}^{2+}$ signals exist,^43^ techniques using fluorescent ${\mathrm{Ca}}^{2+}$ indicators have become the most widely used, and their continuing optimization and development allow real-time monitoring of intracellular ${\mathrm{Ca}}^{2+}$ with high spatial and temporal resolution. It is important to be aware that ${\mathrm{Ca}}^{2+}$ sensors act as ${\mathrm{Ca}}^{2+}$ buffers, potentially altering the dynamics of intracellular ${\mathrm{Ca}}^{2+}$ signaling by binding free ${\mathrm{Ca}}^{2+}$. This should be carefully considered when designing experiments and interpreting data.

### 2.1 Synthetic Chemical Ca^2+^ Sensors

Chemical ${\mathrm{Ca}}^{2+}$ indicators have been widely used to study brain cell activity, including aspects of NVC, owing to their high temporal resolution and sensitivity to rapid changes in intracellular ${\mathrm{Ca}}^{2+}$. These indicators fall into two major types, ratiometric and single-wavelength, each with distinct advantages for imaging ${\mathrm{Ca}}^{2+}$ dynamics (Table 1).

**Table 1:** Selected calcium indicators used in brain imaging.

Type	Family	Sensor	Color	Transgenic/AAVs	1P/2P	Kd (μM)	Examples of applications/notes
**Synthetic sensors**							
		Oregon green 488 BAPTA-1 AM	Green		1P/2P	∼0.17 ^44^	Astrocytes in the neocortex of AD mice;^45^ somatosensory cortex in ${\mathrm{IP}}_{3}R2\text{-}\mathrm{KO}$ mice;^46^ neurons of the rat cerebellar cortex *in vivo*;^47^^,^^48^ cortical neurons and astrocytes in an *in vivo* epileptic rat model^49^
							Fast rise and decay; high signal strength^44^
		X-Rhod-1 AM	Red		1P/2P	∼0.70 ^44^	Neurons and astrocytes from the olfactory bulb, both ex vivo and *in vivo*;^50^ astrocytes in the mouse somatosensory cortex *in vivo*^9^ and *ex vivo*;^51^^,^^52^ cortical astrocytes from an *in vivo* epileptic rat model;^49^ astrocytes from rat hippocampal-neocortical slices^39^
							Fast kinetics^44^
						∼0.60	
		Rhod-2 AM					
		Calcium Green-1	Green		1P/2P	∼0.19 ^44^	Neurons in the mouse motor cortex *in vivo*;^53^ the turtle olfactory bulb;^54^ the rat somatosensory cortex;^55^ rat and mouse olfactory bulb *in vivo*;^56^ neurons and astrocytes from hippocampal brain slices^57^
							Fast kinetics, moderate signal strength^44^
		Fluo-4 AM	Green		1P/2P	∼0.35 ^44^	Neurons in the mouse neocortex *in vivo*;^58^ astrocytes from hippocampal slices,^59^^,^^60^ the rat cerebral cortex *in vivo*;^61^ neurons of hippocampal brain slices;^57^ neurons from Xenopus laevis larvae^62^
							Fast kinetics, strong signal strength^44^
		Fluo-8 AM	Green		1P/2P	∼0.43 ^63^	Astrocytes from the mouse olfactory bulb;^64^ ensheathing cells from the olfactory bulb;^65^ brain slices from the mouse cortex, hippocampus, and cerebellum^66^
							Fast kinetics, high signal strength^67^
		Calcium Ruby Nano	Red		1P/2P	∼0.26 ^68^	Astrocytes from the olfactory bulb in slice and *in vivo* preparations^11^
							Fast kinetics^68^
		Rhod-5N	Red		1P/2P	∼19 ^44^	Mitochondrial calcium in cultured neurons,^69^ co-cultures of cortical or hippocampal neurons and astrocytes^70^
		Cal-520 AM	Green		1P/2P	∼0.32 ^67^	Neocortical neurons in anesthetized mice^71^
							Very fast and very high signal strength^67^
		Cal-590 AM	Red		1P/2P	∼0.56 ^72^	Neurons from the mouse cortex *in vivo*^72^
							Fast kinetics, high signal strength^73^
		Indo-1 AM	Blue/violet		1P/2P^a^	∼0.23 ^44^	Neurons from the neocortex in slice and *in vivo* preparations;^74^ neurons and astrocytes from rat cortical slices,^36^ visual cortex and hippocampus.^75^^,^^76^
							Fast kinetics
		Fura-2 AM	Green		1P/2P^a^	∼0.15 ^44^	Cultured astrocytes from the rat hippocampus, cortex, and cerebellum;^77^ astrocytes from the somatosensory cortex *ex vivo* and *in vivo*^78^
							Moderate to fast kinetics
**Genetically encoded sensors**							
	GCaMPs						
		GCaMP2	Green	Transgenic/AAVs	1P/2P	∼0.15 ^79^	Pyramidal cells in acute cortical brain slices;^80^ neurons in the mouse olfactory bulb and cerebellum *in vivo*;^81^ neurons in the olfactory bulb of rats and mice *in vivo*^56^
		GCaMP3	Green	Transgenic/AAVs	1P/2P	∼0.35 ^82^	Can detect ${\mathrm{Ca}}^{2+}$ signals at the subcellular level with significant temporal resolution. Photobleaching, temperature and pH dependence.
							Astrocytes from visual cortex,^83^ somatosensory cortex;^9^ and other regions;^11^^,^^84^ neurons and astrocytes from the inferior colliculus and auditory cortex;^85^ glial cells from the mouse retina^86^
							Moderate kinetics
		GCaMP5	Green	Transgenic/AAVs	1P/2P	∼0.45 ^82^	The signal-to-noise ratio is better than GCaMP3
							Neurons from striatal slices;^87^ cortical microglia is an ischemic stroke model^88^
							Moderate kinetics
							brighter baseline than GCaMP8
							Mural cells from brain slices and *in vivo*;^89^ neurons and astrocytes from the mouse neocortex *in vivo*;^90^^–^^92^ microglia;^93^ oligodendrocyte precursor cells^94^
		GCaMP6s	Green	Transgenic/AAVs	1P/2P	∼0.14 ^82^	High sensitivity and rapid kinetics, with a
		GCaMP6m	Green	Transgenic/AAVs	1P/2P	∼0.17 ^82^	Microglia in the hippocampus;^95^ neurons from the primary somatosensory cortex and anterolateral motor cortex in mice^96^
		GCaMP6f	Green	Transgenic/AAVs	1P/2P	∼0.38 ^82^	Faster kinetics with a lower detection efficiency.
							Neurons from the mouse olfactory bulb,^97^ excitatory and inhibitory neurons from the somatosensory cortex in an epileptic model,^98^ excitatory neurons from the hippocampus and visual cortex;^99^ neurons in the somatosensory cortex *in vivo* in rats;^25^ astrocytes;^8^^,^^26^ striatal astrocytes^100^
		Lck GCaMP3	Green	Transgenic/AAVs	1P/2P	∼0.15 ^101^	Membrane-tethered.
							Astrocyte ${\mathrm{Ca}}^{2+}$ microdomains from acute hippocampal slices^59^
		Lck GCaMP6f	Green	Transgenic/AAVs	1P/2P	∼0.38 ^82^	Astrocytes in brain slices and *in vivo*;^8^^,^^102^^,^^103^ microglia^93^
		ER-GCaMP6	Green	AAVs	1P/2P	∼150−300 ^104^	Target the ER.
							Astrocytes;^105^ cultured hippocampal neurons^104^
		GCaMP7	Green	Transgenic/AAVs	1P/2P	∼0.15 ^106^	High sensitivity, rapid rise, slow attenuation, highest ΔF/F
							Astrocytes in awake mice during vigilance;^107^ neurons from the mouse somatosensory cortex^108^
		GCaMP8s/m/f	Green	Transgenic/AAVs	1P/2P	∼0.05 ^106^	Greater signal ($F_{\max}/F_{\min}$) but lower fluorescence intensity
							Endothelial cell calcium in retinal tissue *ex vivo*;^109^ endothelial cells from isolated arterioles^110^
						∼0.11	
						∼0.33	
		JRGECO	Red	Transgenic/AAVs	1P/2P	∼0.16 ^111^	Neurons and astrocytes from the prefrontal cortex *in vivo*^112^^,^^113^
		R-CEPIAer	Red	AAVs	1P/2P^a^	∼565 ^114^	Cortical pyramidal neurons, used simultaneously with Mito-RCaMP or CEPIA3mt^115^
		G-CEPIAer	green	AAVs	1P/2P	∼672 ^114^	Cortical and hippocampal astrocytes in brain slices;^116^ astrocytes from the somatosensory cortex *ex vivo* and *in* *vivo*^78^
		CEPIA2mt/3mt	green	AAVs	1P/2P^a^	∼0.16 ^114^	Cortical and hippocampal astrocytes;^116^ cortical pyramidal neurons, used simultaneously with R-CEPIAer^115^
						∼17	
		R-CEPIA3mt/4mt	red	AAVs	1P	∼3.7 ^117^	R-CEPIA3mt has broader dynamic range than R-CEPIA4mt.
						∼27	
							Cultured Hela cells^117^
		Mito-RCaMP	Red	–	1P/2P^a^	∼1.6 ^118^	In cortical pyramidal neurons, used simultaneously R-CEPIAer^115^
							Moderate to fast kinetics
		Mito-GCaMP6f	Green	AAVs	1P/2P	∼0.38 ^118^	Co-cultures of cortical or hippocampal neurons and astrocytes^70^
							Fast kinetics
		YC3.60	Cyan/ Yellow	Transgenic/AAVs	1P/2P	∼0.25 ^119^	Schwann cells, astrocytes, and fine terminal processes of protoplasmic astrocytes in brain slices and *in vivo*: detect both spontaneous and sensory-induced signals; can detect signals within microdomains; exhibit low signal strength *in vivo*;^120^ neurons from acute brain slices, whole-mount retina, and *in vivo* imaging of the olfactory bulb;^121^ pyramidal neurons of the mouse somatosensory cortex^122^
		YC-Nanos	Cyan/Yellow	Transgenic	1P/2P	∼0.05 ^123^	Astrocytes from the somatosensory cortex *in vivo*^123^
		TN-XL	Cyan/Yellow	Transgenic	1P/2P	∼2.5 ^124^	Enhancing the maximal fluorescence change (i.e., 400% change in emission ratio): demonstrated in *Drosophila* and primary hippocampal neurons,^124^ cortical neurons from macaque monkeys *in vivo*^125^
		TN-XXL	Cyan/Yellow	Transgenic	1P/2P	∼0.80 ^126^	*In vivo* imaging of neurons in flies and the mouse visual cortex,^126^ CNiFER implants in rats^127^

a Indicates limited or not optimized for the imaging modality

#### 2.1.1 FRET-based ${\mathrm{Ca}}^{2+}$ sensors

Ratiometric indicators, such as Fura-2 and Indo-1, exhibit a change in excitation or emission spectra upon binding ${\mathrm{Ca}}^{2+}$. This spectral shift enables ratio-based measurements that allow ${\mathrm{Ca}}^{2+}$ concentration to be estimated; it also makes it possible to correct for issues such as variability in dye loading, leakage, photobleaching, and tissue movement—properties that are particularly valuable for *in vivo* imaging.^75^^,^^128^ In their seminal work highlighting the involvement of astrocyte ${\mathrm{Ca}}^{2+}$ in NVC, Zonta and colleagues^36^ used Indo-1 to demonstrate that ${\mathrm{Ca}}^{2+}$ elevations in astrocytes precede vasodilatory responses to neuronal stimulation. Because Indo-1 labels both neurons and astrocytes, the authors had to use morphological criteria and directly inject astrocytes with Lucifer yellow via patch pipette to distinguish them from neurons—a limitation common to all synthetic dyes. Furthermore, the authors noted a limitation in assessing endfoot ${\mathrm{Ca}}^{2+}$ due to inadequate loading of Indo-1.^36^

#### 2.1.2 Single-fluorophore Ca^2+^ sensors

Rhod2-AM,^9^^,^^11^^,^^51^^,^^129^ Fluo-4 AM,^46^^,^^59^^,^^130^ and Oregon Green 488 BAPTA-1AM (OGB1)^46^^,^^130^ are single-wavelength ${\mathrm{Ca}}^{2+}$ indicators. Work using Rhod2-AM or Fluo-4 AM in brain slices further confirmed that astrocyte ${\mathrm{Ca}}^{2+}$ elevations are responsible for stimulus-induced vascular changes.^38^^,^^40^ Interestingly, *in vivo* studies from several laboratories using intensity-based ${\mathrm{Ca}}^{2+}$ indicators reported consistent increases in neuronal activity, alongside sporadic or delayed astrocyte ${\mathrm{Ca}}^{2+}$ signals that often occur after the onset of functional hyperemia in response to sensory stimulation.^9^^,^^46^ However, other studies reported a rapid onset of astrocyte ${\mathrm{Ca}}^{2+}$ transients that preceded vasodilation.^131^^,^^132^ These dyes, characterized by their high ${\mathrm{Ca}}^{2+}$-binding affinity, minimal sequestration within cellular compartments, and broad dynamic range,^133^ are generally brighter and easier to use than ratiometric dyes. They have been widely adopted for studying ${\mathrm{Ca}}^{2+}$ dynamics in brain cells, despite being more susceptible to artifacts caused by photobleaching, uneven dye loading, and variations in tissue thickness.

Both classes of indicators have been instrumental in advancing the study of NVC by enabling real-time visualization of ${\mathrm{Ca}}^{2+}$ dynamics in neurons and astrocytes. Notwithstanding their considerable advantages, chemical ${\mathrm{Ca}}^{2+}$ indicators are not without their limitations, including difficulty loading cells in thick brain slices or *in vivo*, their restriction to acute studies, and their tendency to be compartmentalized. They also suffer from photobleaching, dye leakage, and—importantly—non-specific loading. Notably, in this latter context, most chemical ${\mathrm{Ca}}^{2+}$ indicators are indiscriminately taken up by both neurons and astrocytes, making assessment of cell-type-specific ${\mathrm{Ca}}^{2+}$ dynamics challenging without additional strategies. These experimental modifications include co-loading a morphological dye (e.g., sulforhodamine-101), using parameters such as morphology, electrophysiological properties, or pharmacological responses to distinguish cell types, or applying microinjection and/or patch-clamp techniques to selectively label specific cells.^36^^,^^46^^,^^75^^,^^134^ All of these alternatives are time-consuming and invasive, and none provides true cell-type selectivity. For example, sulforhodamine-101 can aid in labeling neurons, but only in older animals, and microinjections associated with patch-clamp procedures can damage the cell. There are exceptions, and studies have reported that Rhod2-AM and Fluo-4 AM are preferentially taken up by astrocytes and not neurons.^51^^,^^61^^,^^135^ It should be noted that these studies measured relatively slow ${\mathrm{Ca}}^{2+}$ waves (i.e., 1-s scale),^51^^,^^61^^,^^136^ and dyes such as Fluo-4 are dim in the absence of free ${\mathrm{Ca}}^{2+}$, so the fluorescent signal would be brighter in cells with higher resting ${\mathrm{Ca}}^{2+}$.

Some of these ${\mathrm{Ca}}^{2+}$ indicators also show modest loading of other cell types, including vascular smooth muscle and endothelial cells. This modest loading of some ${\mathrm{Ca}}^{2+}$ indicators presents a particular challenge when studying astrocyte endfeet ${\mathrm{Ca}}^{2+}$ signals in the context of NVC, where accurate measurement of astrocyte endfeet ${\mathrm{Ca}}^{2+}$ responses can be hindered by the presence of endfeet ensheathing vessels and the simultaneous loading of ${\mathrm{Ca}}^{2+}$ indicators in both endfeet and vascular cells.^36^ Reeves and colleagues^130^ reported that, despite effectively sampling the soma, bulk loading of Fluo-4 AM alone failed to sample the majority of astrocyte processes. The complementary approach of bulk loading a ${\mathrm{Ca}}^{2+}$ indicator with morphological “maps” was shown to improve the detection of ${\mathrm{Ca}}^{2+}$ transients by ∼80%.^130^ Given the heterogeneity of ${\mathrm{Ca}}^{2+}$ signals across astrocyte subcellular compartments,^137^ ${\mathrm{Ca}}^{2+}$ signals from one compartment cannot reliably serve as a surrogate for another in attempting to fully understand astrocyte ${\mathrm{Ca}}^{2+}$ dynamics and their role in NVC. Other indicators have been developed with improvements that make them more suitable for (1) multi-photon ${\mathrm{Ca}}^{2+}$ imaging, such as ${\mathrm{Ca}}^{2+}$ rubies, a family of BAPTA-based red fluorescent ${\mathrm{Ca}}^{2+}$ indicators;^138^ (2) studying mitochondrial ${\mathrm{Ca}}^{2+}$, such as rhod-5N^139^; (3) or achieving greater sensitivity and signal-to-noise ratio, such as Cal-520, Cal-590, and Cal-630.^73^ Their ease of loading, broad spectrum of ${\mathrm{Ca}}^{2+}$ affinities and fluorescence properties, versatility, and wide availability have made chemical ${\mathrm{Ca}}^{2+}$ indicators a popular choice, especially for cultured cell studies.

### 2.2 Genetically Engineered Ca^2+^ Sensors

The development of protein-based ${\mathrm{Ca}}^{2+}$ indicators has revolutionized our approach to assessing ${\mathrm{Ca}}^{2+}$ signals, and the integration of protein-based ${\mathrm{Ca}}^{2+}$ indicators with Cre-lox technology has significantly advanced our understanding of cell-type–specific ${\mathrm{Ca}}^{2+}$ dynamics.^59^^,^^102^^,^^140^^–^^142^ Available genetically encoded ${\mathrm{Ca}}^{2+}$ indicators (GECIs) are composed of amino acids that form a ${\mathrm{Ca}}^{2+}$-binding protein consisting of a ${\mathrm{Ca}}^{2+}$-binding domain (e.g., calmodulin) fused to fluorescent proteins (FPs). Once expressed, these protein-based ${\mathrm{Ca}}^{2+}$ sensors localize to the cytosol without leaking into non-expressing cells or sequestering into organelles, making them suitable for chronic studies.^126^^,^^143^

#### 2.2.1 FRET-based ${\mathrm{Ca}}^{2+}$ sensors

Among FRET-based GECIs are members of the Cameleon family and Troponin C family (Table 1). Cameleons, the first FRET-based ${\mathrm{Ca}}^{2+}$ indicators, have paved the way for many subsequent FRET-based indicators.^140^^,^^144^ These GECIs consist of tandemly fused blue- or cyan-emitting mutants of green fluorescent protein (GFP), calmodulin, the calmodulin-binding peptide M13, and an enhanced green- or yellow-emitting FP.^140^ Atkin et al.^120^ constructed a transgenic mouse line expressing the yellow Cameleon, YC 3.60, under control of a human S100β promoter that specifically expresses this GECI in Schwann cells and astrocytes, although some expression was detected in other glial cell types such as polydendrocytes (NG2 cells), microglia, 2’,3’-cyclic nucleotide 3’-phosphodiesterase (CNP)-positive oligodendrocyte progenitor cells, and some large motor neurons in the brain stem. This indicator is not only expressed in cytoplasmic cellular compartments, but it is also found in fine terminal processes of protoplasmic astrocytes.^120^ Although attempts to express YC3.60 in both astrocytes and neurons under the control of the ubiquitous β-actin promoter were not promising,^145^ the performance of YC3.60 specifically targeted to astrocytes proved to be faithfully reliable for imaging in both brain slices and *in vivo*.^120^ In this case, YC3.60 showed good signal-to-noise ratios for both spontaneous and sensory-induced ${\mathrm{Ca}}^{2+}$ signals, and for detection not only in individual cells but also within microdomains.^120^ Although S100β-driven YC3.60 demonstrated significantly improved utility in assessing astrocyte ${\mathrm{Ca}}^{2+}$ dynamics compared with previously developed Cameleons, exhibiting enhanced signal-to-noise ratios, larger dynamic range, and better photostability and brightness, they are not widely used, especially for *in vivo* imaging, owing to their limited expression level and thus low signal strength *in vivo*.^120^^,^^145^

A new generation of ${\mathrm{Ca}}^{2+}$ indicator was developed using a variant of troponin C (TnC)—a specialized ${\mathrm{Ca}}^{2+}$ sensor in the skeletal and cardiac muscle as a ${\mathrm{Ca}}^{2+}$-binding moiety in place of calmodulin.^124^^,^^126^ This FRET-based, TnC–derived indicator, termed TN-XL, is thought to minimally disturb cell biochemistry, given that troponin, unlike calmodulin, is not a part of a signal transduction pathway. Continuing optimization of the first-generation sensor has significantly improved troponin-based FRET ${\mathrm{Ca}}^{2+}$ sensors, increasing their overall signal strength and sensitivity, making them pH insensitive in the physiological range, and providing reliable signals in response to small changes in neuronal electrical activity.^124^^,^^126^ These new sensors have been used in various applications, including the use of cell-based neurotransmitter fluorescent engineered reporters (CNiFERs) to study cell-to-cell signaling by monitoring cytosolic ${\mathrm{Ca}}^{2+}$ changes in cultured cells in response to acetylcholine release,^127^ and for chronic *in vivo* imaging to study sensory-evoked neuronal activity in the visual cortex.^126^ TN-XXL is expressed at high levels, allowing visualization of subcellular structures such as dendritic spines with negligible photobleaching.^126^ Although TN-XXL performance seemed comparable to that of OGB-1 in several respects, OGB-1-AM detected more stimulus-responsive cells and generated a stronger maximal peak response compared with TN-XXL,^126^ suggesting room for improvement of this type of sensor. Even though the performance of TN-XXL may lag that of synthetic ${\mathrm{Ca}}^{2+}$ indicators such as OGB-1 AM, this ratiometric ${\mathrm{Ca}}^{2+}$ indicators not only allow repeated measurements of ${\mathrm{Ca}}^{2+}$ signals and thus facilitates chronic *in vivo* studies, but it also helps to alleviate movement artifacts—a challenge in awake *in vivo* imaging as well as in contracting cells such as vascular smooth muscle cells. Although there are clear advantages with these indicators, such as ratiometric signal, genetically encoded, and compatibility with live imaging in tissues and *in vivo*, these indicators have not been used in other cell types such as vascular smooth muscle cells, pericytes, or endothelial cells.

#### 2.2.2 Single-fluorophore GECIs

The mono-fluorescent protein ${\mathrm{Ca}}^{2+}$ indicator family includes GCaMPs,^102^ Camgaroos,^146^ Pericams,^147^ GECOs,^148^ and CatchERs^149^ (Table 1). These ${\mathrm{Ca}}^{2+}$ indicators are typically fusion proteins comprising a circularly permuted FP and the ${\mathrm{Ca}}^{2+}$ binding protein, calmodulin. Although other mono-fluorescent protein indicators have been tested and used in various applications,^146^^–^^148^ GCaMPs are the most frequently used single-fluorophore GECIs. Since its inception with GCaMP1, developed by Nakai and colleagues,^150^ the GCaMP series has grown to include eight members, each existing in various forms. The original GCaMP1 contained either an enhanced GFP or a circularly permuted GFP,^150^ which are known to yield greater fluorescent changes than FRET-based ${\mathrm{Ca}}^{2+}$ probes.^140^^,^^145^^,^^146^ The integration of advanced single-fluorophore GECIs and the Cre-loxP systems has revolutionized our approach to cell-type–specific noninvasive acute and chronic ${\mathrm{Ca}}^{2+}$ imaging and advanced our understanding of numerous fields, including NVC. GCaMP6 sensors, more advanced GCaMP series variants, have been commonly adopted to study NVC in recent years.^8^^,^^26^^,^^97^^–^^99^ With its greater signal-to-noise ratios, GCaMP6 has been shown to reliably detect single action potentials in pyramidal neuron somata and orientation-tuned synaptic ${\mathrm{Ca}}^{2+}$ transients in individual dendritic spines^82^^,^^151^ and to detect a significantly higher fraction of active neurons than either GCaMP5 or OGB1-AM.^82^ Using *Pdgfrb*-CreERT2 x LSL-GCaMP6s to monitor ${\mathrm{Ca}}^{2+}$ signals in mural cells of the mouse somatosensory cortex, distinct ${\mathrm{Ca}}^{2+}$ dynamics were observed and characterized in vascular smooth muscle cells, ensheathing pericytes, and capillary pericytes.^89^ The ${\mathrm{Ca}}^{2+}$ signals from these specifically identified mural cells highlight the heterogeneity of ${\mathrm{Ca}}^{2+}$ dynamics and differential contributions of mural cells to vascular tone. In particular, the characterized capillary pericytes’ ${\mathrm{Ca}}^{2+}$ dynamics support the notion that capillary pericytes contribute to basal blood flow resistance and slow modulation of blood flow throughout the brain.^152^ Among all the cell types investigated using GCaMPs to measure ${\mathrm{Ca}}^{2+}$ signals, astrocytes emerged as the most widely studied. The use of GCaMPs has advanced our understanding of how dynamic astrocyte ${\mathrm{Ca}}^{2+}$ transients are within different subcellular compartments.^8^^,^^26^^,^^100^^,^^153^ There are three forms of GCaMP6: GCaMP6s, GCaMP6m, and GCaMP6f, with slow, medium and fast kinetics, respectively. The faster kinetics of GCaMP6f come with a lower detection efficiency compared with GCaMP6s and GCaMP6m, which are more sensitive to ${\mathrm{Ca}}^{2+}$ than OGB-1 AM.^82^ As more imaging tools to study ${\mathrm{Ca}}^{2+}$ dynamics become available, our ability to appreciate the diversity of spatiotemporal patterning of ${\mathrm{Ca}}^{2+}$ signals expands.^59^^,^^102^^,^^137^

These ${\mathrm{Ca}}^{2+}$ signals can originate from the extracellular space, intracellular stores, or a combination of both. The complexity of astrocyte ${\mathrm{Ca}}^{2+}$ signals, especially within ${\mathrm{Ca}}^{2+}$ microdomains and their role in NVC, has gradually come into focus, thanks largely to the development of Lck-GCaMP6f—a membrane-tethered version of GCaMP6 that can be used to target astrocytes with Cre-lox technology.^102^ The design of this indicator allows it to be tethered to the plasma membrane, enabling the detection of ${\mathrm{Ca}}^{2+}$ signals specifically at the cell surface. To our knowledge, Lck-GCaMP6f has mostly been adopted to study ${\mathrm{Ca}}^{2+}$ responses in astrocytes.^8^^,^^83^^,^^102^^,^^103^^,^^154^^,^^155^ A study by Stobart and colleagues^155^ using Lck-GCaMP6f showed a fast onset of astrocyte ${\mathrm{Ca}}^{2+}$ microdomain signals, which the authors suggested could be involved in initiating vasodilation, although direct measurements of vascular responses were not performed. Another study has proposed that astrocyte ${\mathrm{Ca}}^{2+}$ amplifies functional hyperemia when neuronal activation is prolonged.^8^ Although Lck-GCaMP6f is not as widely used as cytosolic GCaMP6f owing to its unique localization to the plasma membrane and detection only of ${\mathrm{Ca}}^{2+}$ microdomains, its characteristics suggest that it can complement the performance of cytosolic GCaMP6. In addition, Lck-GCaMP6, although it has not been used to explore ${\mathrm{Ca}}^{2+}$ dynamics in vascular cells, giving its unique properties, Lck-GCaMP6 can potentially be used to explore ${\mathrm{Ca}}^{2+}$ sparklets, a highly localized ${\mathrm{Ca}}^{2+}$ influx through L-type ${\mathrm{Ca}}^{2+}$ channels in vascular smooth muscle cells, which regulate local and global intracellular ${\mathrm{Ca}}^{2+}$ and modulate vascular function.^156^^,^^157^

An improved version of GCaMP6, GCaMP6f^82^ and jGCaMP7,^158^ can detect single action potentials, population activity, and changes in small synaptic compartments;^159^^,^^160^ however, they exhibit reduced sensitivity compared with GCaMP6s. GCaMP8, the newest version of GCaMP GECIs, exhibits improved kinetics without compromised sensitivity or brightness. There are three variants: jGCaMP8s (fast rise, slow decay, and sensitive), jGCaMP8f (fast rise and fast decay), and jGCaMP8m (fast rise and medium decay).^106^ jGCaMP8 has been used to study mouse neuronal ${\mathrm{Ca}}^{2+}$ in the visual cortex, where it showed fluorescence rise times nearly 10-fold faster than those of previously developed GCaMPs.^106^ A transgenic mouse line expressing GCaMP8 under the transcriptional control of cadherin 5 (*Cdh5*^BAC^-GCaMP8) has been reported to detect a hierarchy of endothelial ${\mathrm{Ca}}^{2+}$ signals, including brief, low-amplitude events, slowly propagating responses, and long-lasting, large compound signals.^110^^,^^161^^,^^162^ These findings implicate the involvement of endothelial ${\mathrm{Ca}}^{2+}$ and cell-to-cell communication in mediating retrograde signals that contribute to the downstream pathways of NVC.^110^^,^^163^

To better understand ${\mathrm{Ca}}^{2+}$ dynamics, researchers have increasingly moved toward assessing ${\mathrm{Ca}}^{2+}$ signals in different organelles, such as the endoplasmic reticulum (ER) and mitochondria. ER-GCaMP6f, designed to measure signaling near the ER, was constructed using the ER-targeting motif of cytochrome p450 to anchor the sensor to the cytosolic side of the ER membrane.^105^ This differs from previously designed ER-intraluminal GCaMP probes that measure activity on the luminal side.^114^ ER-GCaMP6f detects ${\mathrm{Ca}}^{2+}$ signals generated by ER membrane-resident receptors that go undetected with Lck-GCaMP6.^105^ Because the ER in astrocytes occupies both the soma and processes, measuring ${\mathrm{Ca}}^{2+}$ signals near the ER may offer insights into process-level signaling—an area of astrocyte ${\mathrm{Ca}}^{2+}$ dynamics that remains poorly understood. Iino’s group developed a family of GECIs known as ${\mathrm{Ca}}^{2+}$-measuring organelle-Entrapped Protein IndicAtors (CEPIAs) to measure intraorganellar ${\mathrm{Ca}}^{2+}$, including ER and mitochondrial ${\mathrm{Ca}}^{2+}$.^114^ In particular, work by Kanemaru and colleagues^117^ introduced a red fluorescent CEPIA variant optimized for measuring mitochondrial ${\mathrm{Ca}}^{2+}$ concentrations. They targeted the sensor to the mitochondria by adding a mitochondrial localization signal sequence to the CEPIA variants’ coding sequences.^117^^,^^164^ These CEPIAs exhibit a large dynamic range and strong brightness, enabling visualization of heterogeneous ${\mathrm{Ca}}^{2+}$ dynamics in subcellular mitochondrial domains.^117^ They also provide additional benefits for multiplex imaging.

Compared with FRET-based ${\mathrm{Ca}}^{2+}$ sensors, single-fluorophore GECIs are more user-friendly because of their larger, single-wavelength intensiometric responses. They require a single wavelength for excitation and emission, making it possible to image multiple parameters. However, a major drawback of single-fluorophore GECIs is their lack of quantifiability. Because these intensiometric single-fluorophore GECIs are mostly used to detect all-or-none responses by neurons, their advantages are generally thought to outweigh the need for quantification. However, as we move forward, especially to examine ${\mathrm{Ca}}^{2+}$ signals from non-neuronal cells such as astrocytes, endothelial cells, and vascular smooth muscle cells, where ${\mathrm{Ca}}^{2+}$ within microdomains contributes to the overall signaling pathway, quantification of ${\mathrm{Ca}}^{2+}$ signals becomes more important.

## 3 Voltage Sensors

For decades, electrical activity in both excitable and non-excitable cells has been assessed using conventional electrophysiological methods, including intracellular recordings, extracellular recordings, the patch-clamp technique, and multielectrode arrays. Although these methods have been reliable and have substantially enriched our understanding of the electrophysiological behavior of neurons, smooth muscle cells, and other cell types, they do come with certain limitations and challenges. For example, the stability of intracellular recording can be difficult to maintain, and the procedure can be invasive, potentially affecting cell function; extracellular recordings have limited spatial resolution and lack detailed information about the intracellular processes; and the patch-clamp technique is labor-intensive, low-throughput, and technically demanding, requiring a high level of competence to achieve reliable results. Although ${\mathrm{Ca}}^{2+}$ imaging has transformed our ability to assess neuronal activity, deconvolution of ${\mathrm{Ca}}^{2+}$ signals into underlying voltage changes remains challenging.

### 3.1 Organic Voltage-sensitive Dyes

In the mid-1970s, the pioneering work from Cohen’s laboratory screening a series of commercially available dyes led to the identification of the merocyanine class of dyes possessing voltage-sensitive optical properties.^165^ Subsequently, a series of potentiometric dyes was developed. These typically belong to two classes: electrochromic dyes, which have low sensitivity but are rapid enough to track action potentials,^166^ and oxonol dyes, which have larger fractional fluorescence responses to voltage but respond slowly.^167^ Slow-response dyes, such as tetramethylrhodamine methyl ester, capable of measuring mitochondrial membrane potential, have also been developed.^168^ The use of these synthetic, organic voltage indicators has flourished in recent years, especially in the neuroscience field, where they are typically used for neural mapping.^169^^–^^172^ Although their application in the true context of NVC remains rather limited, an increasing number of studies suggest their potential for exploring NVC. Sigler and colleagues^173^ used RH1692 to record changes in cortical membrane potential *in vivo* in the mouse somatosensory cortex, where they found that sensory-evoked depolarization could rapidly redistribute to a perfused but nonfunctional region following a local stroke. Similarly, voltage-sensitive dyes have been used in conjunction with other imaging modalities to show that the state of neuroglial energy consumption does not necessarily determine regional blood flow via vasoactive metabolite production.^174^ In addition to neurons and astrocytes, researchers can utilize these voltage-sensitive dyes to study other cell types of the neurovascular unit, including vascular smooth muscle cells, pericytes, and endothelial cells. Although voltage-sensitive dyes have proven their utility, they suffer several drawbacks, including low signal-to-noise ratio, limited spatial resolution, uneven tissue penetration, and sensitivity to background staining; importantly, these dyes lack high cell-type specificity.^175^ Many voltage-sensitive dyes produce small fluorescence changes (e.g., 0.1%) in response to relatively small changes in membrane potential (e.g., 10 mV).^176^ As with most fluorescent dyes, voltage-sensitive dyes often show poor tissue penetration, limiting their distribution to deeper regions; light scattering and absorption in thick tissue further hinder imaging at depth. These synthetic dyes have been extensively reviewed elsewhere.^177^

### 3.2 Genetically Encoded Voltage Indicators

To combat some of the drawbacks presented by voltage-sensitive dyes, researchers have devoted significant efforts to the development of genetically encoded voltage indicators (GEVIs). The first GEVI, named FlaSh, was developed by Siegel and Isacoff. It was a modified GFP fused to a voltage-sensitive $K^{+}$ channel in such a way that voltage-dependent rearrangements in the $K^{+}$ channel induce changes in the fluorescence of the GFP.^178^ This probe exhibited a maximal fractional fluorescence change of 5.1%, making it comparable to some of the best voltage-sensitive dyes. However, its kinetics were slow, preventing its use in tracking action potentials; it also failed to traffic adequately to the plasma membranes of mammalian cells.^178^ This development was followed by a series of GEVIs with various modifications designed to increase their response kinetics, membrane localization, and sensitivity.^175^ More than 30 GEVIs have been developed to date.^179^ Table 2 lists representative examples of voltage-sensitive dyes and GEVI. These indicators are typically categorized into three types: (1) voltage-sensitive fluorescent protein-based, (2) microbial rhodopsin-based, and (3) chemogenetic (hybrid).

**Table 2:** Selected voltage indicators used in brain imaging.

Type	Family	Sensor	Color	Transgenic/AAVs	1P/2P	Kinetics	Examples of applications/notes
**Synthetic sensors**							
	RH	RH-1691	Red		1P/2P^a^	Fast (ms)^180^	Neurons from the barrel cortex in awake, behaving mice;^169^^,^^181^ neurons from the somatosensory cortex in anesthetized rats^182^
		RH-1692	Red		1P/2P^a^	Fast (ms)^183^	Neurons from cortical brain slices and *in vivo*^184^^,^^185^
		RH-795	Red		1P/2P^a^	Fast (ms)^183^	Neurons from the somatosensory cortex in anesthetized rats;^186^ mouse brain stem slices^187^
		RH-414	Red		1P/2P^a^	Fast (ms)^188^	Rat neocortical slices;^188^ isolated cerebellum^189^
	ANEPPS	Di-4-ANEPPS	Red-orange		1P/2P^a^	Very fast (∼μs)^190^	Neurons from cortical, hippocampal, and primary visual cortex brain slices of rats.^190^^–^^192^
		Di-8-ANEPPS	Red-orange		1P/2P^a^	Very fast (∼μs)^193^	Endothelial cells from Isolated rat middle cerebral arteries;^194^ cultured rat microglia;^195^ neurons from mouse hippocampal brain slices^193^
**Genetically encoded sensors**							
		ArcLight	Green	Transgenic/AAVs	1P/2P^a^	Slow (∼10−50 ms)^196^	Mouse olfactory sensory neuron *ex vivo*^197^ and *in vivo*;^198^^,^^199^ mouse hippocampal neurons;^200^ neurons from cortical brain slices;^190^^,^^201^ astrocytes from cortical brain slices^202^
							High ΔF/F^196^
	ASAP	ASAP1	Green	AAVs	1P/2P^a^	Moderate (∼5−10 ms)^196^	Developed and tested in primary rat hippocampal neurons;^203^ cultured pyramidal neurons^204^
							Lower signal than ArchLight^196^
		ASAP2s/2f/2s-Kv	Green	Transgenic/AAVs	1P/2P	Moderate (∼4−7 ms)^196^	Developed and tested in cultured rat hippocampal neurons;^205^ Drosophila optic lobes *in vivo*^206^
						Faster (∼2−3 ms)	
						Moderate	
		ASAP3	Green	AAVs	1P/2P	Fast (∼0.5−1 ms)^207^	Hippocampal and cortical slices and in cortex and hippocampus in awake *in vivo*^207^
		ASAP5	Green	Transgenic^b^/AAVs	1P/2P	Very fast (<1 ms)^208^	Developed and tested in cultured rodent neurons and human stem-cell-derived neurons^208^
							Brightest GEVIs and the fastest member of the ASAP family^208^
		JEDI-2P	Green	AAVs	1P^a^/2P	Very fast (∼0.5 ms)^208^	Developed and tested on cortical neurons from awake, behaving mice^209^
		PostASAP	Green	–	1P/2P	–	Developed and tested on dendrites and spines of cortical neurons *in vivo*^210^
		Archon 1/2	Red	AAVs	1P/2P^a^	Very fast ∼0.3−0.5 ms)^211^	Neurons from cortical brain slices^190^; SomArchon—Archon 1 localized to the soma;^212^ tested in visual and motor cortical, hippocampal, and striatal neurons in brain slices and in awake *in vivo*;^212^ astrocytes from cortical brain slices^202^
		FlicR1	Red	AAVs	1P/2P^a^	Fast (∼1−3 ms)^213^	Developed and tested on rat hippocampal neurons^213^
		QuasAr1/QuasAr2/QuasAr3/paQuasAr3	NIR	AAVs	1P	Ultra-fast (∼0.1 ms)^196^	Developed and tested on neurons from the hippocampus and olfactory bulb in brain slices and *in* *vivo*^214^^,^^215^
							QuasAr1 is dim, others are brighter^196^
	Ace	Ace-mNeon/2	Green/Red	Transgenic^b^/AAVs	1P/2P^a^	Fast (∼1−3 ms)^196^	Developed and tested for neural spiking in brain slices and dendritic activation in awake mice and flies;^216^ employed to study PV interneurons in awake mice^217^
		pAce	Red-shifted	Transgenic^b^/AAVs	1P	Fast (∼1 ms)^218^	Reverse response-polarity variant of Ace; developed and tested in a several neuron types, including VIP+, SST^+^ neurons, in awake mice^218^
		${\text{Voltron}}_{525}$	Red-NIR	AAVs	1P/2P	Very fast (<1 ms)^213^	Developed and tested on hippocampal PV neurons and visual cortex pyramidal neurons *in vivo* including some awake imaging;^219^ mouse cortical interneurons^211^
		${\text{Voltron}}_{549}$	Orange-Red	AAVs	1P/2P	Very fast (<1 ms)^219^	Developed and tested in cultured rat neurons and mouse brain slices^219^
		ChiVSFP	Green	Transgenic/AAVs	1P/2P^a^	Slow (∼10−20 ms)^190^	Neurons from cortical brain slices^190^

a Indicates limited or not optimized for;

b indicates only available in flies

#### 3.2.1 Voltage-sensitive domain–based voltage sensors

The first voltage-sensitive domain-based voltage sensors utilized the voltage-sensing domain (VSD) from the voltage-sensing phosphatases (VSP) from *Ciona intestinalis* (Ci-VSP) containing an insertion of super-ecliptic pHluorin, a noncanonical FP, converting this FP to a voltage-sensitive FP.^220^^,^^221^ GEVIs such as ArcLight and Marina, the first generation of this class, display high sensitivity to membrane potential, and their kinetics and brightness are sufficient to track action potentials.^220^ Implementing a strategy similar to that used for designing GECIs, researchers inserted circularly permuted GFPs into Ci-VSDs to yield the new GEVIs, ElectricPk, and the ASAP (accelerated sensor of action potentials) series. These GEVIs exhibit exceptional kinetics and good trafficking to the plasma membrane,^222^ overcoming a challenge encountered in first-generation GEVIs. GFPs are the most widely used components for both GECIs and GEVIs; however, increasing demand to image deeper and utilize multiple fluorescent probes in a single experiment has made red fluorescent proteins more desirable. One such red fluorescent voltage indicator protein is FlicR1, which retains all of the features of ASAP.^213^ Members of the FlicR series have fast kinetics but a small dynamic range and dim fluorescence.^213^ Similar to synthetic voltage sensors, these GEVIs have not been widely implemented to study NVC. However, studies such as that by Carandini et al.,^223^ which used VSFP-Butterfly, a GEVI capable of detecting not only spiking activity but also subthreshold inputs, in conjunction with a custom projection method to separate voltage and hemodynamic signals using wide-field imaging, demonstrate the potential of GEVIs for exploring NVC. Given that vascular smooth muscle cells, pericytes, and endothelial cells do not generate all-or-none electrical activities such as action potentials, or large and dynamic such as synaptic potentials in neurons, but rather slow and small electrical dynamics,^220^^,^^224^ it is understandable that voltage imaging techniques have not been widely implemented in these cases. However, with advancements in GEVI, such as the recently developed voltage indicator rEstus, which was reported to be ultrasensitive and optimized for non-excitable cells,^225^ researchers can now optimize and employ these sensors for cells other than neurons. One emerging area of interest involves pericytes, a relatively controversial non-excitable cell type.^152^^,^^226^^–^^228^ Pericytes have been shown to maintain a more depolarized resting membrane potential than vascular smooth muscle cells, express functional voltage-dependent ${\mathrm{Ca}}^{2+}$ channels,^224^ and exhibit distinctive current density and kinetics profile of potassium channels, including Kir, Kv1, and ${\mathrm{BK}}_{\mathrm{Ca}2+}$.^229^ Given their proposed roles in regulating vascular tone^152^ and controlling microvascular blood flow and distribution,^230^^,^^231^ further investigation into the electrical properties of pericytes is both warranted and timely.

#### 3.2.2 Rhodopsin-based voltage sensor

The second class of GEVIs was designed based on the natural electrochromism of bacteriorhodopsin. Bacteriorhodopsin, a light-driven proton pump, has been widely used in optogenetics to manipulate cellular activity (activate or silence) using light, enabling a wide range of applications in neurobiology.^9^^,^^12^

##### Directly fluorescent rhodopsin-based

Rhodopsin-based voltage sensors such as those from the Archaerhodopsin (Arch) system were designed based on a similar concept but repurposes the inherent light-driven outward proton pump activity of Arch to allow detection of changes in membrane potential.^232^^,^^233^ Although these sensors were shown to detect changes in membrane potential, they are quite dim, requiring high light power to achieve sufficient fluorescence. Furthermore, because these opsins can respond to wavelengths typically used for imaging, they can perturb cell membrane potential.^233^ Cohen’s group reported that introducing a single amino acid mutation in Arch deactivates the proton-pumping mechanism, and the resulting mutant variant, Arch(D95N), can effectively sense voltage changes without inducing a photocurrent.^232^^,^^233^ Additional screens of a series of Arch mutants yielded QuasAr1, QuasAr2, and Mero-6, all of which showed improved brightness and reduced photocurrent, but less than optimal plasma membrane trafficking in eukaryotic cells.^234^ Many studies have used indicators from this family to study neuronal activity across various brain regions, including the cerebral cortex and hippocampus, in both brain slices and *in vivo*.^190^^,^^200^ Interestingly, several studies have used these indicators for chronic voltage imaging in the olfactory bulb.^198^^,^^199^ To our knowledge, no studies have directly used these indicators to investigate NVC. However, imaging approaches from these studies could be adapted for NVC studies.

##### FRET-based

The next generation of rhodopsin-based GEVIs, designed based on the concept of FRET, fused a bright FP (e.g., mNeon) as the FRET donor to a voltage-sensitive opsin (e.g., Ace) as the FRET acceptor.^216^^,^^218^ The two sensors designed using this concept, Ace-mNeon and VARNAM, show a decrease in fluorescence—green and red, respectively—during depolarization. The same group identified the corresponding reverse response-polarity variants, pAce and pAceR, which exhibit an increase in fluorescence with depolarization (i.e., during an action potential).^218^ These indicators can be used simultaneously to image multiple neuron types with sub-millisecond kinetics, allowing detection of individual spikes and fast spike trains with ∼0.2  ms accuracy.^216^ The authors reported the use of these indicators to examine electrical activity in several neuron types, including somatostatin-expressing interneurons and vasoactive intestinal peptide-expressing interneurons in the visual cortex and hippocampus of running mice.^218^ These studies pave the way for further exploration of the roles of somatostatin-expressing interneurons and vasoactive intestinal peptide-expressing interneurons, both of which have been implicated in NVC.^235^ By combining the standard (e.g., Ace-mNeon) and reverse response-polarity (e.g., pAce) variants, researchers can leverage the opposite response polarities of these sensors to simultaneously image voltage changes in two distinct neuron types using a single fluorescence channel.^218^ Even though GEVIs of this new generation offer better performance than previously designed GEVIs, they are not free of drawbacks. They are technically complex; they show limitations in imaging depth. They also require a sparse labeling strategy to limit signal overlap. Fidelity of subthreshold membrane potential measurements with these indicators is limited by frequency dependence and noise, making it difficult to reliably detect small voltage changes.^218^ Work performed with these GEVIs used widefield one-photon epifluorescence for *in vivo* imaging, with recordings restricted to superficial cortical layers. Although widefield imaging is often favored for real-time voltage monitoring due to its simplicity and ability to capture broad spatial activity, it does not fully capture the precision enabled by patterned light delivery techniques, which are commonly employed to enhance spatial resolution and signal fidelity in these superficial regions. It should be noted that patterned light delivery systems, such as those using galvo/resonant scanning, digital micromirror devices, are technically sophisticated and often require complex optical alignment and calibration to achieve high signal-to-noise ratio targeting in voltage imaging applications. A pair of red-shifted, opsin-based GEVIs, Cepheid1b and Cepheid1s, was recently reported to have a wider dynamic range, improved brightness, and photostability in HEK293T cells compared with the previously reported red-shifted GEVIs, VARNAM, VARNAM2, Ace2N-7aa-mScarlet, and AceC81-mScarlet-I1.4.^216^^,^^218^^,^^236^ When used to measure action potentials in cultured rat hippocampal neurons, they also showed good expression and membrane localization in the soma and dendrites; similar results were obtained when these sensors, introduced into mice via AAV infection, were used to image various brain regions in brain slices.^236^

#### 3.2.3 Chemogenetic indicators

Continuing research efforts seek to develop sensors with high photostability and low phototoxicity through optimization of various classes of GEVIs. Among these are chemogenetic voltage indicators—hybrid sensors that combine a genetically encoded protein tag (e.g., HaloTag) with a synthetic, voltage-sensitive fluorescent dye. In these indicators, a protein tag is genetically targeted to a specific cell type, and the synthetic dye is applied exogenously, binding covalently to the tag. This approach uses genetic targeting to ensure cell-type–specific labeling, an emphasis that distinguishes these systems from synthetic dyes, which are used to provide brighter, faster kinetics and greater photostability.^213^ The same group that developed the latter indicators reported a family of indicators called Voltron that, when expressed in neurons, can detect fast membrane potential changes with high fidelity, making them compatible with *in vivo* imaging as well as multiplex and dual-polarity imaging applications.^237^ Because this approach requires dye application, it may not be suitable for deep and chronic imaging, although the authors showed good delivery *in vivo.*

Voltage sensors face several challenges that have caused them to lag behind ${\mathrm{Ca}}^{2+}$ sensors in terms of development and widespread adoption. Although a growing number of GEVIs and organic voltage indicators have been reported, their utility remains uncertain in many experimental contexts, including NVC. However, several indicators show potential for use in studying NVC. Many of these sensors are not fully characterized, partly due to the lack of standardized benchmarking protocols, the technical complexity of voltage imaging, and reproducibility issues. Although many voltage sensors perform well in the lab where they were developed, they have not always proven to produce reliable results in other settings.

## 4 cAMP Sensors

Similar to ${\mathrm{Ca}}^{2+}$, which is a vital second messenger involved in almost every physiological process, cyclic adenosine monophosphate (cAMP) is a universal second messenger that regulates a variety of biological functions ranging from gene transcription and synaptic plasticity to development and learning and memory.^238^^–^^241^ Although the technology for studying ${\mathrm{Ca}}^{2+}$ using optical imaging is significantly more advanced than that for imaging cAMP, it has become increasingly possible to visualize cAMP events. In the brain, activation of G-protein–coupled receptors (GPCRs) by neurotransmitters such as norepinephrine, dopamine, and acetylcholine triggers signaling pathways that regulate neuronal excitability and plasticity involving the generation of either ${\mathrm{Ca}}^{2+}$ or cAMP, depending on which receptor subtype is activated.^242^ Downstream of cAMP are four effectors: cAMP-dependent protein kinase (PKA), exchange proteins activated by cAMP (EPAC), cAMP-gated channels, and Popeye-domain–containing (POPDC) proteins.^243^^–^^246^ cAMP signaling exhibits both cell-specific and subcellular compartmentalized properties,^247^^,^^248^ such that a given stimulus can elevate or decrease intracellular cAMP levels in distinct cell types or within different subcellular compartments, leading to diverse biological outcomes.^249^^,^^250^ This compartmentalization is further underscored by the spatial and temporal regulation of cAMP’s major effector, PKA, which is itself modulated differently in various cells.^251^ As a result, detecting cAMP dynamics with high spatial and temporal resolution remains a significant challenge. Both synthetic and genetically encoded cAMP indicators have been developed, and each offers distinct advantages and disadvantages.

### 4.1 Synthetic cAMP Sensors

Similar to the design concepts used for organic ${\mathrm{Ca}}^{2+}$ indicators and voltage-sensitive dyes, cAMP sensors have been designed based on changes in fluorescence intensity or FRET that occur upon binding to cAMP. The most common organic cAMP indicators are FRET-based. With this design, a donor and acceptor fluorophore are linked via a cAMP-binding domain, often a cyclic nucleotide-binding domain (CNBD) derived from PKA, EPAC, or other cAMP-binding proteins. Upon binding of cAMP, the cAMP-binding domain undergoes a conformational change that alters the distance or orientation between donor and acceptor fluorophores, influencing the FRET signal as discussed above. The first cAMP sensor, FlCRhR, was designed using recombinant PKA catalytic (PKA-C) and regulatory (PKA-R) subunits tagged with the organic dyes, fluorescein and rhodamine, respectively. This development leveraged the fact that PKA-C and PKA-R subunits dissociate from each other in the presence of cAMP.^252^ The intensity of the resulting FRET signal was inversely correlated with the concentration of cAMP in the cell, such that increased cAMP concentrations resulted in decreased FRET.^252^

Synthetic cAMP indicators are highly versatile, and similar to other organic sensors, can be easily modified to tune their sensitivity, specificity, and fluorescence properties. They are also capable of detecting cAMP dynamics in real-time with high spatial and temporal resolution, making them useful for both *in vitro* and *in vivo* experiments. However, they are not yet commonly adopted for studying brain activity. Similar to synthetic organic ${\mathrm{Ca}}^{2+}$ and voltage sensors, their main limitation is their lack of cell-type specificity. There are also additional concerns about poor cell permeability, which may require modification of the transfection techniques used for cellular uptake.

### 4.2 Genetically Encoded cAMP Sensors

Genetically encoded sensors have transformed the study of cAMP signaling by enabling real-time cell-specific monitoring of both cAMP and its downstream effector, PKA, in living cells and tissues. To date, more than 80 such sensors have been developed for *in vitro* and *in vivo* applications.^253^ These sensors fall into three broad categories: (1) sensors based on cAMP binding, (2) sensors that detect PKA subunit dissociation, and (3) sensors that monitor PKA-mediated phosphorylation events. They can be further subdivided according to their detection strategy—either FRET-based or fluorescence intensity-based—each with its own strengths and limitations (see Massengill et al. for a detailed review).^253^ Table 3 summarizes key examples of genetically encoded cAMP sensors.

**Table 3:** Selected genetically encoded cAMP indicators.

Type	Family	Sensor	Color	Transgenic/AAVs	1P/2P	Kd (μM)	Examples of applications notes
		Pink Flamindo	Red	AAVs	1P/2P^a^	∼3.2 ^254^	Developed and tested in astrocytes from the cerebral cortex *in vivo*;^254^ hippocampal neurons;^255^ astrocytes in awake mice during vigilance^107^
		G-Flamp1	Green	AAVs	1P/2P	∼2.2 ^256^	Developed and tested in cortical neurons;^256^ HED293T cells^257^
							Fast kinetics: G-Flamp2 is faster than G-Flamp1
		G-Flamp2					
						∼0.7	
	AKAR	AKAR	Cyan/Yellow	Transgenic	1P/2P^a^	–	Neurons from the nucleus accumbens *in vivo*;^258^ D1 and D2 receptor-expressing neurons in the dorsal striatum;^259^ cultured hippocampal neurons;^260^ rat cultured astrocytes;^261^ pyramidal neurons from mouse cortex^262^
							AKAR3EV is the fastest among the 3 variants
		AKAR2					
		AKAR3EV					
	Epac	ICUE	Cyan/Yellow	Transgenic	1P/2P^a^	∼1−2	Cultured neurons;^263^ hippocampal neurons;^260^ mouse^264^^,^^265^ and human arterial myocytes^265^
		Epac1-camps	Cyan/Yellow		1P/2P^a^	∼2.7	Brain slices from the mouse cortex, hippocampus, and cerebellum;^66^ rat cultured astrocytes;^261^ pyramidal neurons from mouse cortex^262^
						∼0.30	
		Epac2-camps300					
		cAMPFIRE	Cyan/Yellow	–	1P/2P	∼1−2	Developed and tested in hippocampal slices;^266^
							Fast kinetics (<1 s)
		Camper	Cyan/Yellow	Transgenic	1P/2P	∼1−2	Developed and tested in pyramidal neurons of the hippocampus and cortex;^267^ aortic vascular smooth muscle cells;^268^ neurons from the striatum^269^
							Fast kinetics (∼1 s)
		CUTie	Cyan/Yellow	AAVs	1P/2P^a^	∼1.7	Developed and tested in cardiac myocytes;^270^ aortic vascular myocytes^268^

a Indicates limited or not optimized for;

b indicates only available in flies

#### 4.2.1 Sensors detecting PKA subunit dissociation

The first genetically encoded PKA-based cAMP sensor, PKAc-S65T+PKArII-EBFP, was a FRET-based sensor designed to monitor cAMP-induced dissociation of the PKA catalytic and regulatory subunits. This approach mirrored earlier synthetic sensors but used genetically encoded FPs instead of chemical dyes.^271^ This approach laid the groundwork for the development of both dissociation-based and phosphorylation-based cAMP reporters. However, sensors based solely on subunit dissociation have seen limited development compared with other sensor classes.

#### 4.2.2 Sensors monitoring PKA-mediated phosphorylation events

Phosphorylation-based sensors detect PKA activity by reporting phosphorylation events catalyzed by PKA rather than directly measuring cAMP levels. The most widely used family in this category is the A kinase activity reporter (AKAR) family, first developed in 2001, with numerous improved variants since reported.^272^^–^^276^ Variants from this class are highly adaptable and compatible with live-cell and *in vivo* imaging—factors that contribute to their widespread use—and have recently been used to generate transgenic mice. These sensors incorporate a PKA substrate sequence, a phosphoamino acid-binding domain, and a FRET pair of fluorescent proteins that transduce phosphorylation into a ratiometric fluorescence signal. One of the most advanced AKAR variants is AKAR3EV, which has been incorporated into transgenic mouse models.^277^ Similar to AKAR3EV, Booster-PKA, another phosphorylation-based sensor with red-shifted excitation and emission wavelength, was specifically designed to detect PKA-mediated phosphorylation events.^275^ Because phosphorylation-based reporters such as AKARs and Booster-PKA do not directly measure cAMP levels, they can be confounded by phosphatase activity or other signaling inputs that modulate PKA independently of cAMP. In addition, PKA itself is subject to spatiotemporal regulation that may diverge from cAMP dynamics.^262^^,^^275^^,^^278^ Most studies using these indicators to examine cAMP or PKA activity have focused on neuromodulatory effects and associated behaviors, primarily in neurons and, to a lesser extent, in astrocytes.^259^^,^^261^^,^^262^ For example, Castro et al. found that type 4 phosphodiesterase plays distinct roles in different cellular domains of pyramidal cortical neurons. In the cytosol, it can act as a brake, attenuating cAMP/PKA signaling in response to β-adrenergic stimulation.^262^ Although these studies did not directly study NVC, their findings suggest potential relevance, especially as an increasing number of works point to astrocyte-mediated, ${\mathrm{Ca}}^{2+}$-independent signaling pathways and the involvement of neuromodulators such as serotonin, acetylcholine, and norepinephrine in NVC.^26^^,^^84^^,^^235^^,^^279^

#### 4.2.3 Sensors based on cAMP binding

To directly visualize cAMP dynamics, researchers developed a separate class of genetically encoded sensors based on single-polypeptide FRET constructs incorporating a cAMP-binding domain. These fall into two main designs: EPAC-based sensors, using either full-length or truncated forms of EPAC,^280^ and sensors based on the cAMP-binding domain of the PKA regulatory subunit (PKA-R).^281^

EPAC-based sensors, such as ICUE and EPAC1-camps, have been widely adopted to study cAMP dynamics in cultured cells owing to their robust FRET responses and large dynamic range, resulting from substantial conformational changes upon cAMP binding.^280^^,^^282^ However, full-length EPAC proteins are large and difficult to package into viral vectors.^282^ Smaller variants constructed from only the cAMP-binding domain of PKA-R have been developed, offering easier delivery at the cost of a smaller conformational change and a correspondingly reduced FRET response and diminished dynamic range.^281^^,^^283^

Recent refinements have led to the development of the EPAC-S series of sensors (e.g., EPAC-SH188, EPAC-SH159, and EPAC-SH189), which offer notable improvements on EPAC-based FRET sensors in terms of dynamic range, signal-to-noise ratio, brightness, photostability, and reduced aggregation.^283^ A few years back, the Zaccolo Lab developed a new cAMP sensor called CUTie (cAMP Universal Tag for imaging experiments), featuring a unique design in which the acceptor FP (i.e., YFP) is inserted into the middle of the cyclic nucleotide-binding domain from PKA RIIβ, whereas the donor FP (i.e., CFP) is fused to the C-terminus.^270^ This configuration reduces the influence of adjacent sequences when the sensor is tagged to targeting domains or proteins. A key advantage of CUTie over Epac-based cAMP sensors is its consistent dynamic range across different subcellular compartments, making it suited for targeted applications. Although CUTie has not yet been used in neurons, it has been successfully used in vascular cells.^268^ Navid et al. reported that nicotine impairs β-adrenergic cAMP synthesis in vascular smooth muscle cells. These findings highlight CUTie potential utility for studying cAMP dynamics within the neurovascular unit.

Starting by screening existing genetically encoded cAMP sensors, Massengill and colleagues engineered three enhanced variants from the best performer and designated them cAMP Fluorescence Imaging Reporters based on EPAC (cAMPFIREs).^266^ These variants offer several advantages, including compatibility with both ratiometric and fluorescence lifetime imaging, which enable detection of cAMP dynamics in brain tissue and allow for longitudinal measurements. These sensors also exhibit increased sensitivity, demonstrating the ability to detect cAMP dynamics at physiologically relevant concentrations and resolve subcellularly compartmentalized cAMP signals.^266^ More recently, the group of Kirill A. Martemyanov developed a transgenic mouse line, known as CAMPER, which conditionally expresses an EPACA1-based FRET sensor in tissues of interest.^267^ The authors took advantage of the latest generation probe composed of an mTurquoise donor and a tandem cp173Venus-Venus acceptor, flanking the cAMP-binding domain from EPAC1. By expressing the CAMPER sensor specifically in neurons, the authors demonstrated how distinct inputs generate unique cAMP signatures in different neuronal populations.^267^ The CAMPER mouse has also been used to interrogate cAMP signaling in vascular smooth muscle cells of intact arteries, where it was shown that chronic nicotine exposure impairs cAMP signaling, leading to reduced vasorelaxation.^268^ These findings highlight the versatility of the CAMPER mouse as a tool for probing how diverse signaling pathways integrate complex information to modulate NVC.

#### 4.2.4 Beyond FRET-based sensors

Despite their power, FRET-based sensors have known limitations, including relatively low signal-to-noise ratios and the need for sophisticated imaging setups. Consequently, there has been growing interest in developing fluorescence intensity-based genetically encoded sensors. Such efforts have led to the development of PKA-SPARK, sapphireAKAR, blueAKAR, and RAB-AKARev.^253^ Although many of these variants have not been extensively characterized, two notable exceptions are R-FlincA and Pink Flamindo. R-FlincA exhibits a relatively large dynamic range ($\Delta F/F_{0}$ of ∼2.5),^254^^,^^284^ whereas Pink Flamindo has been successfully applied together with two-photon imaging *in vivo* to study astrocyte cAMP levels during prolonged wakefulness, revealing that prolonged periods of vigilance are accompanied by a sustained increase in cAMP.^107^

Both synthetic organic and genetically encoded cAMP indicators have revolutionized our study of cAMP signaling, offering real-time, high-resolution measurements in living cells and tissues. Although organic sensors are useful for their flexibility and ease of use, genetically encoded indicators offer long-term, noninvasive, and cell-specific monitoring of cAMP dynamics. However, challenges remain in improving sensitivity, reducing background noise, and achieving precise localization within subcellular compartments.

## 5 Conclusion

Sensors used for optical imaging are broadly divided into synthetic organic dyes and genetically encoded indicators. These sensors can be further subdivided into two categories: single-fluorophore and multi-fluorophore. Single-fluorophore sensors typically report fluorescence intensity changes, whereas multi-fluorophore sensors are typically ratiometric or FRET-based. Genetically encoded indicators offer several advantages, including high specificity, sensitivity, non-invasivity, real-time monitoring, and—importantly—cell-type specific labeling. Although these features make these indicators suitable for *ex vivo* and *in vivo* imaging, they suffer from photobleaching, which limits their use in chronic studies. Single-fluorophore sensors, which can be excited and detected using a single wavelength, are typically easier to use and are suitable for multiplex imaging with different reporters (e.g., ${\mathrm{Ca}}^{2+}$ sensors). Because they only contain a single FP, these sensors also tend to be smaller in size, facilitating their packaging into viral vectors. Fluorescence intensity measurements are also sensitive to movement artifacts, changes in cell volume, and sensor concentration, which can be problematic in *in vivo* imaging, where breathing, hemodynamics, and movement are unavoidable. By contrast, ratiometric sensors are less sensitive to variations in concentration or movement, but FRET-based sensors require two color channels and are less amenable to sensor-multiplexing strategies. Optical imaging for ${\mathrm{Ca}}^{2+}$, voltage, and cAMP has advanced significantly over the past few decades. Although ${\mathrm{Ca}}^{2+}$ indicators remain the most extensively developed, characterized sensors of these sensors, and widely adopted to study brain function, voltage, and cAMP sensors are showing increasingly promising results, with continuing improvements in sensitivity, signal-to-noise ratio, and localization capacity.

Notwithstanding critical roles of second messengers and voltage sensors in understanding neural function and NVC, there has been a growing and renewed interest in the role of extracellular signaling molecules and neuromodulators in brain function. This is particularly evident in the context of studying NVC. This emerging focus has driven the need for the development of novel biosensors. Several types of sensors have been developed or are in development to address this need, including a series of GPCR-activation-based sensors (GRAB), which assess spatiotemporal dynamics of neurotransmitters such as ATP,^285^ dopamine,^286^ and norepinephrine.^287^ Each sensor offers unique strengths and limitations, and although a comprehensive review of these sensors is beyond the scope of the current work, they should be discussed in the future as these sensors undergo further testing, validation, and adoption for specific applications.

Considering the incredible rate at which the array of available sensors is growing, choosing an appropriate sensor for a given application can be overwhelming, particularly in the face of inconsistent or context-dependent literature findings. Moreover, finding the perfect sensor that meets all desirable experimental criteria—large dynamic range, high signal-to-noise ratio, fast kinetics, high photostability, minimal phototoxicity, minimal interference with cellular functions, and multiplex imaging options—may be more of an aspirational goal than a realistic one. Ultimately, researchers will need to make informed trade-offs based on the specific needs of their experimental design. It is important to note that the optimal choice of fluorescent sensor often depends on factors such as targeted cell type, temporal resolution, and imaging modality. Detailed guidance on sensor selection tailored to these parameters is beyond the scope of this review.

## Data Availability

Data sharing is not applicable to this article, as no new data were created or analyzed.
